# Epstein Barr Virus-induced Antiphospholipid Antibodies Resulting in Splenic Infarct: A Case Report

**DOI:** 10.7759/cureus.4119

**Published:** 2019-02-22

**Authors:** Salman Khan, Shakir Saud, Imran Khan, Sheela Prabhu

**Affiliations:** 1 Internal Medicine, Guthrie Clinic/Robert Packer Hospital, Sayre, USA; 2 Family Medicine, Rutgers New Jersey Medical School, Newark, USA; 3 Internal Medicine, North Shore University Hospital, Hempstead, USA

**Keywords:** epstein barr virus, ebv, apa, antiphospholipid, splenic infarct

## Abstract

Splenic infarction is a rare cause of abdominal pain. We herein reported a unique case of a 30-year-old male patient who developed a splenic infarct during the acute phase of Epstein-Barr virus (EBV)-associated infectious mononucleosis (IM) and was subsequently found to have the presence of antiphospholipid antibodies (APA).

## Introduction

Epstein-Barr virus (EBV) has been associated with infectious mononucleosis (IM) as its common infection. With its insidious onset of fever, sore throat, swollen posterior cervical lymph nodes, and fatigue, diagnosis can be difficult to determine early in its course [1]. This case study has described a finding of antiphospholipid antibodies (APA), rarely found in the literature. Further investigation could help develop the role of APAs in EBV. 

## Case presentation

A 30-year-old male with no comorbidities presented to the emergency room in February 2018 with complaints of headache, fatigue, dry cough, and abdominal pain that started three days prior to admission. Apart from occasional alcohol consumption, his past medical history was unremarkable with no history of surgery or trauma.

On presentation, vitals were within normal limits with the exception of a temperature of 102 °F and his physical exam results were as follows: he appeared to be in moderate distress. Skin was jaundice; his abdominal exam was notable for diffuse abdominal tenderness with hepatosplenomegaly. Lower extremities revealed traced edema. Initial laboratory studies revealed a white blood count of 4.4 K/uL with lymphocytic predominance, hemoglobin of 16 g/dL, platelets 150 K/uL, aspartate aminotransferase (AST) 116 U/L, alanine aminotransferase (ALT) 119 U/L, and creatinine level of 1.1 mg/dL. Hepatitis and human immunodeficiency virus (HIV) panels were negative. Herpes simplex virus (HSV) and cytomegalovirus (CMV) were negative. A presumptive diagnosis of infectious mononucleosis was made and confirmed by serological and polymerase chain reaction (PCR). The EBV viral capsid antigen IgM antibody was >160 (normal <0.9); viral capsid antigen IgG antibody was negative, EBV early antigen IgG was 1.54 (normal <0.9), and the EBV nuclear antigen IgG was negative.

**Figure 1:**
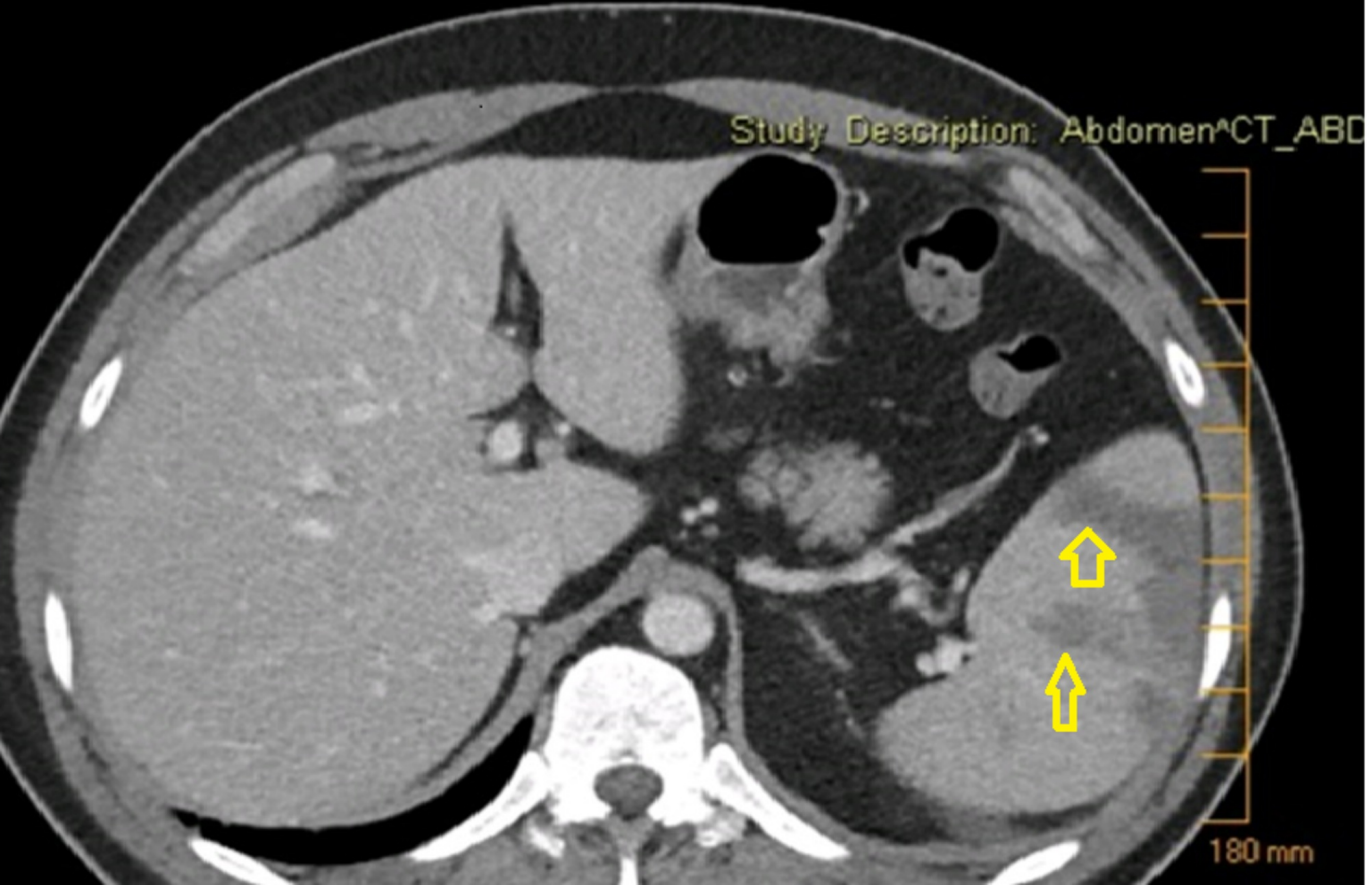
CT abdomen with arrows pointing to splenic infarction CT: computed tomography

On day three of admission, acute worsening of abdominal pain with shortness of breath complicated the hospital course. Repeat labs were white blood corpuscle (WBC) count of 17.1 K/uL with lymphocytic predominance, hemoglobin 7.8 g/dL, platelets 667 K/uL, AST 332 U/L, and ALT 146 U/L. A computed tomography (CT) was performed, which revealed a wedge infarct of the spleen (Figure 1). APAs were sent at that time and were positive. He was transferred to the intensive care unit and started on bilevel positive airway pressure (BiPAP) and continuous renal replacement therapy (CRRT) due to anuria. His clinical status improved with supportive therapy; a repeat CT scan showed improvement of splenic infarcts and he was discharged 27 days later. Repeat APA testing six weeks later was negative. 

## Discussion

EBV is an HSV that is spread by intimate contact between a susceptible person and an infected person. EBV has a wide clinical spectrum with most cases of IM being self-limiting with no long-term sequelae. Interestingly, EBV infection has been correlated with several types of malignancies such as B cell lymphoma, T cell lymphoma, Hodgkin's lymphoma, and nasopharyngeal carcinoma [1]. The majority of infections are subclinical and asymptomatic. Approximately 90% to 95% of adults are EBV seropositive [2].

EBV-induced infectious mononucleosis is often seen in younger adults with the most common presentation being sore throat, fever, malaise, lymphadenopathy, and pharyngitis. The presence of splenomegaly, palatal petechiae, and posterior cervical adenopathy is highly suggestive of IM [3]. The virus can be transmitted in salivary secretions for up to six months following initial exposure [4]. Rare complications include splenomegaly, hepatomegaly, jaundice, and splenic infarct [5].

The most common lab finding is lymphocytosis. Monospot testing is the initial diagnostic test of choice but has a high false negative up to 25% in acute infection [5]. IgM antibody directed against the viral capsid antigen has high sensitivity and specificity and is present at the onset of clinical symptoms due to the long incubation period and can confirm the diagnosis of acute IM. IgG viral capsid antigens antibodies are present for life following EBV infection [6].

Splenic infarction is a rare complication observed in a small subset of patients with acute IM and is managed non-operatively in the majority of cases [7]. The number of splenic infarctions associated with IM is unknown and the mechanism is yet to be elucidated. There have been several hypotheses including demand ischemia secondary to spleen enlargement, a transient hypercoagulable state that results in low perfusion to the spleen, and elevated APAs that initiate an autoimmune-mediated infarction via thrombosis [8]. In addition to the present case, other documented cases of EBV infection are being associated with a transient induction of APAs. Van Hal et al. report a case of splenic infarction in a patient with acute IM who was found to have elevated APAs that normalized after resolution of acute illness [9]. Further studies will be needed to establish the hypothesis of the correlation between APAs and splenic infarction in patients with acute IM.

## Conclusions

The role of APAs that were found positive during this acute illness is yet to be established. We inferred the transient rise of APAs found during the acute phase has a correlation with EBV. Going forward, further research could be geared toward the role of APAs in EBV. 
